# Active trachoma two years after three rounds of azithromycin mass treatment in Cheha district Gurage zone, Southern Ethiopia

**DOI:** 10.1186/1471-2431-13-199

**Published:** 2013-12-01

**Authors:** Fisseha Admassu, Samson Bayu, Abebe Bejiga, Bemnet Amare

**Affiliations:** 1 Department of Ophthalmology, University of Gondar, College of Medicine and Health Sciences, Gondar, Ethiopia; 2 Department of Ophthalmology, Addis Ababa University, Addis Ababa, Ethiopia; 3 Department of Medical Biochemistry, University of Gondar, College of Medicine and Health Sciences, Gondar, Ethiopia

**Keywords:** Active trachoma, Mass treatment, Azithromycin, Ethiopia

## Abstract

**Background:**

Azithromycin mass distribution was given to residents of Gurage zone Cheha district in 2004, 2005 and 2006 for three consecutive years with more than 90% coverage. The effect of treatment in the study community was not yet determined. The present study was therefore designed to assess the effect of azithromycin on the prevalence of active trachoma two years after three rounds of mass treatment of the community at Cheha district, Gurage zone.

**Methods:**

A multistage stratified cluster random survey was employed to determine the prevalence of active trachoma among children aged 1 to 9. Selected children were examined for trachoma using the simplified WHO grading system and their households were assessed for trachoma risk factors.

**Results:**

This survey demonstrated that the prevalence of active trachoma in the study community was 22.8% (95% CI 18.24% - 27.36%) that was lower than that of Southern Nations, Nationalities, and People's Regional prevalence (33.2%) in 2006. Only 27.6% (95% CI 25.7% - 30.1%) of the study population had a safe and clean water supply, whereas 42.7% (95% CI 39.8% - 46.2%) of the visited households had simple pit latrines.

**Conclusion:**

This survey demonstrated that despite repeated mass oral azithromycin distributions, the prevalence of active trachoma was still high. Therefore, the other components of the SAFE strategy such as fly control program, improving the water sources, measures to improve face washing and construction of utilizable latrines that are being implemented through the health extension package have to be integrated with mass azithromycin treatment to eliminate active trachoma in the district.

## Background

Trachoma is a chronic infectious keratoconjunctivitis caused by serotypes A, B, Ba and C of the bacterium *Chlamydia trachomatis*[1,2]. It is the world’s leading cause of preventable blindness [3]. Sixty three million people suffer from active trachoma infection, 7.6 million have trachomatous trichiasis and nearly 10 million people are visually impaired or irreversibly blind as a result of trachoma [3]. The burden of this disease falls disproportionately on poor rural communities, predominantly in Sub-Saharan Africa [4].

World Health Organization (WHO) called to eliminate blinding trachoma by the year 2020 through SAFE strategy [5]. SAFE strategy is a comprehensive public health approach which combines treatment, Surgery (to correct advanced stages of the disease) and Antibiotics (azithromycin to treat infection in individuals**),** with prevention, Facial cleanliness (to reduce transmission of trachoma**)** and Environmental improvement **(**through increased access to clean water and improved sanitation) [6]. Previous studies have shown that in the short term, mass antibiotic distribution can dramatically decrease the prevalence of ocular strains of Chlamydia in villages. Current WHO guidelines recommend 3 annual mass distributions [7].

Azithromycin mass distribution was given to residents of Gurage zone Cheha district for three consecutive years through the ORBIS international program in collaboration with the zonal health bureau. First round was distributed from December 1–10, 2004 with 90% coverage, second round from December 1–10, 2005 with 92% coverage and third round from December 1–10, 2006 with 93% coverage. Baseline estimates and projections of active trachoma for the districts before azithromycin distribution was greater than 40% [unpublished observation]. As part of SAFE strategy implementation, each Kebeles (the lowest political administrative unit in Ethiopia) of the district have trained health extension worker who screen community members for active trachoma and trichiasis, and refer them to the nearby health centers. They also educate the community on personal hygiene, latrine construction and utilization, and proper animal waste disposal. There are also billboards posted by government and non government organization that provide information on mode of transmission, prevention and treatment of trachoma in local language.

Though different studies have shown that community-wide treatment with oral azithromycin markedly reduces *C. trachomatis* infection and clinical trachoma in endemic areas [8,9], the effect of treatment in the study community is not yet determined.

Therefore this research was conducted with a general objective of assessing the effect of azithromycin mass treatment on the prevalence of active trachoma two years after three rounds of mass treatment of the community at Cheha district, Gurage zone that will help for evidence based planning in the future.

## Methods

### Study design and setting

A community-based cross sectional survey was conducted Cheha district found in Gurage zone, in Southern Nations, Nationalities and People Region of Ethiopia 185 Kms south west of Addis Ababa from September 1, 2008 to September 30, 2008. This district consisted of 42 kebeles with a total population of 175,597, projected for the year 2008 out of which children aged 1 to 9 years accounted for 56,194 [10]. The main sources of income are subsistence agriculture and trade. The major part of the district (71%) has a middle land climatic condition, 20% High land and the rest 9% low land with the altitude range of 1200 m to 2600 m and annual rainfall ranging from 800 – 1200 mm [10]. The district has one hospital, four health centers, one health station and 37 health posts that makes 62% physical health service coverage in the year 2007. There are two ophthalmic nurses and four integrated eye care workers who give eye care services at the district. All of the 83 health extension workers in the district were trained by ORBIS on SAFE strategy and help the community on trachoma specific control interventions such as fly control program, building and utilization of latrine and measures to improve face washing.

### Sample size and sampling procedure

The sample size of the study population, children aged 1 to 9 years who resided in the study district, was estimated by using single population proportion formula, [n = (Z α/2)^2^ / p (1-p)] [11]. The following assumptions were made: 95% confidence, 5% margin of error, design effect of 2, and 62.6% prevalence rate from previous studies [12]. Computing with the above formula and 10% of contingency gives a total sample size of 792. The required number of clusters was determined by dividing the calculated sample size by the cluster size (60 children in one cluster) resulting in 13 clusters. After calculating the sampling interval, the 13 clusters were selected from 10 kebeles of the district. In each selected cluster, compact segment sampling method was employed to collect the data.

### Data collection

A pre-tested and structured questionnaire was used to guide for the systematic data collection process and findings were recorded on the forms. Clinical evaluation for trachoma follicles (TF) (defined as the presence of five or more follicles in the upper tarsal conjunctiva) was used to evaluate the response of active trachoma to azithromycin [13]. All selected children were assessed for active trachoma by the principal investigator who had 3 years of experience in trachoma grading using WHO trachoma simplified grading system with binocular examination loupe (2.5 times magnification) and torch light [14]. The children were also assessed for facial uncleanness that was defined by the presence of ocular discharge, nasal discharge and/or flies on the face. Backyards were visited for availability of waste disposal and latrine, and their utilization by the household members.

### Ethical considerations

The study protocol was approved by the Research Ethics Committee of the department of ophthalmology of both University of Gondar and Addis Ababa University. A support letter from the zonal and district health offices was obtained. The purpose of the study was explained and verbal consent from their parents (care takers) was obtained. All children and parents (care takers) who were diagnosed to have active trachoma were given tetracycline ointment to be applied twice daily for six weeks and those with trichiasis and other ocular problems were referred to respective health institution for management.

### Data analysis

Data were entered and analyzed using SPSS version 15 statistical package (SPSS, Inc., Chicago, IL, USA). The analysis part contains descriptive and inferential statistics. Statistical significance was determined by P-value < 0.05.

## Results

A total of 768 children aged 1 to 9 years (with a 97% coverage of the sample size) that included 386 (50.3%) males and 382 (49.7%) females participated in the survey. The mean age of the study group was 6.79 years (Table 1). Out of the 768 children included in the study, 93 (12.1%) didn’t receive azithromycin in the past; whereas 86 (11.2%) had received only once, 86 (11.2%) had received twice and 503 (65.5%) received three times.

**Table 1:** Socio-demographic and environmental variables, and azithromycin treatment history of children aged 1–9 years at Gurage zone Cheha district in October 2008, n = 768

**Variable**		**Active trachoma (TF)**			**Chi square test**
		**Yes n (%)**	**No n (%)**	**Total n (%)**	
**Sex**	Male	93 (12.1)	298 (38.8)	391 (50.9)	P = 0.502
	Female	82 (10.7)	295 (38.4)	377 (49.1)	
	Total	175 (22.8)	593 (77.2)	768 (100)	
**Age**	1	3 (0.4)	16 (2.1)	19 (2.5)	P = 0.002
	2	6 (0.8)	19 (2.50)	25 (3.3)	
	3	18 (2.35)	18 (2.35)	36 (4.7)	
	4	24 (3.1)	8 (1.0)	32 (4.1)	
	5	37 (4.85)	11 (1.45)	48 (6.3)	
	6	39 (5.1)	70 (9.1)	109 (14.2)	
	7	25 (3.2)	131 (17.1)	156 (20.3)	
	8	14 (1.8)	194 (25.2)	208 (27.0)	
	9	9 (1.2)	126 (16.4)	135 (17.6)	
	Total	175 (22.8)	593 (77.2)	768 (100)	
**Past Azithromycin treatment**	Never	53 (6.9)	40 (5.2)	93 (12.1)	P = 0.001
	Once	57 (7.4)	29 (3.8)	86 (11.2)	
	Two times	42 (5.5)	44 (5.7)	86 (11.2)	
	Three Times	23 (3.0)	480 (62.5)	503 (65.5)	
	Total	175 (22.8)	593 (77.2)	768 (100)	

**Figure 1:**
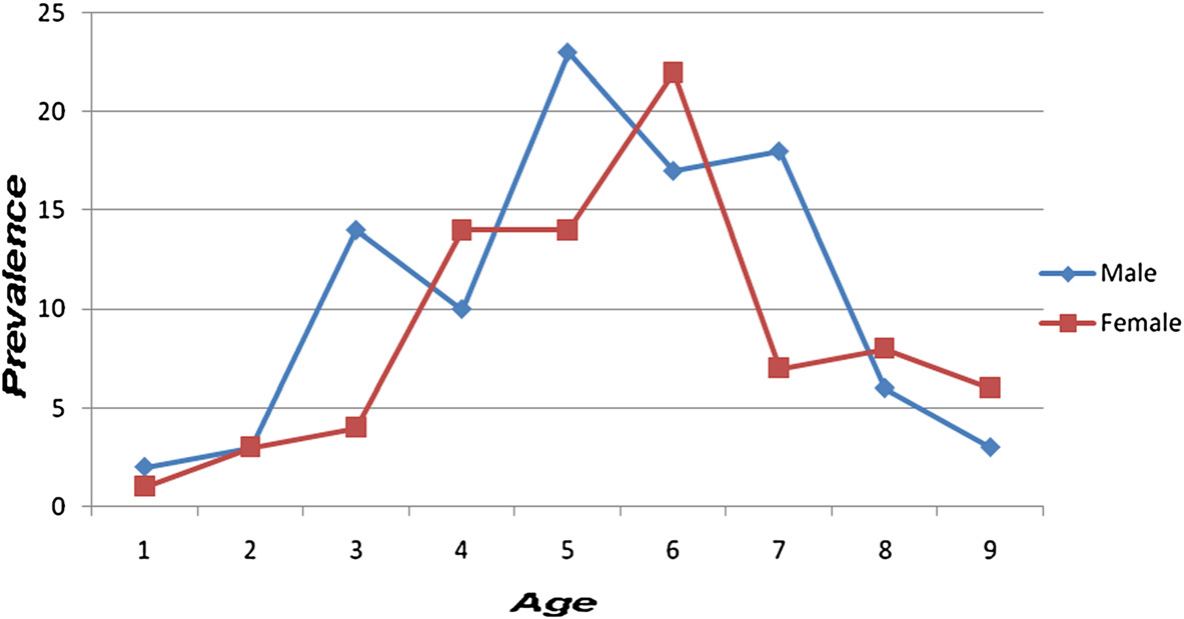
Prevalence of active trachoma by age and sex in children aged 1–9 years at Gurage zone Cheha District in October 2008.

**Figure 2:**
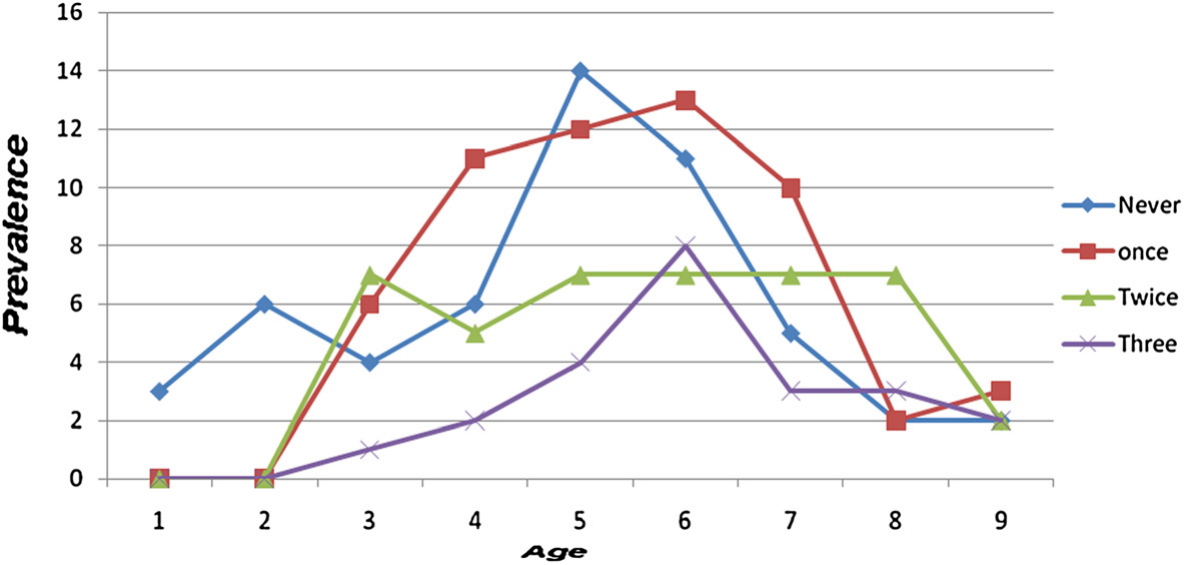
Prevalence of active trachoma by frequency of azithromycin treatment history in children aged 1–9 years at Gurage zone Cheha District in October 2008.

In this study, we found a total of 175 (22.8%) (95% CI 18.24% - 27.36%) children had active trachoma with a slight male preponderance; that is 96 (54.9%) were males. The highest of prevalence trachoma was in the age group 2 to 6 years (Figure 1). Two hundred and seventy six children (35.9%) were having unclean face with flies around their faces and eyes, eye and nasal discharge at the time of the survey. Out of the 49 children who had never received azithromycin in the past, 12 (24.5%) had active trachoma while 28 (47.5%) of children who received the drug one time, 54 (51.4%) of children who received the drug two times and 81 (14.6%) of children who received the drug three times had active trachoma (Figure 2). Three hundred sixteen (41.1%) of the children were found out to have scared tarsal conjunctiva.

Out of the visited ten Kebeles only three had a improved water sources (tape water and protected wells) that accounted for 212 [27.6% (95% CI 25.7% - 30.1%)] of the study population. The rest of the district people used rivers, streams and well as source of water without any treatment. Three hundred and twenty eight [42.7% (95% CI 39.8% - 46.2%)] of the visited households had simple pit latrines, that were made by lying two logs of wood over a pit with wide gap in between the logs and almost all did not have lid to cover, whereas the rest of the population used open fields. It was observed that none of the visited households had proper solid waste and animal disposal. Almost all disposed animal waste in their back yards where they have false banana plantation.

## Discussions

Although active trachoma have not been eliminated from this district after three rounds of community-wide treatment with oral azithromycin, the finding of this survey demonstrated that the overall prevalence of active trachoma in the study area was lower than that of Southern Nations, Nationalities, and People's Regional prevalence ( 33.2%) in 2006 [12]. In line with this, studies from different parts of the country reported that mass treatments with oral azithromycin markedly reduce *C. trachomatis* infection and clinical trachoma in endemic areas [14,15]. In spite of the fact that the prevalence of active trachoma was decreasing in the area, active trachoma is still a disease of public health interest.

Despite the efforts of community health extension workers and other nongovernmental organization to implement the SAFE strategy in the community, the prevalence of active trachoma is still very high. A possible explanation for this might be that there is no adequate water supply in the major part of the community. Furthermore, 47.8% of the community lacks functional latrine and almost all the community dispose animal waste product open field. Another possible explanation might be that mass azithromycin treatment may not be integrated with health promotion through health education on primary eye care, personal and environmental hygiene of the Districts. Recent evidence suggests that all the A, F, E components of the SAFE strategy have independent protective effects against active trachoma [16].

The chance of getting active trachoma was lower for children who had received azithromycin three times than twice or once (odds ratio of 3.2, 2.0 and 1.2, respectively). This finding, supported by many other reports [14,17-21], emphasizes that repeated doses of azithromycin are important for reduction and elimination of the infection.

The current study found that the prevalence of active trachoma is highest in the age range of 3 to 6 years. The higher prevalence of trachoma among these age groups may be explained by the fact that young children are dependent on their families for their personal hygiene. Spending several hours playing on the ground (exposing them to dirt that attracts flies to them) could also put them at risk.

The SAFE strategy anticipates the use of antibiotics and surgery only as short-term interventions that are delivered through the health services. Azithromycin is expensive but easily administrable, safe and effective drug for treatment of trachoma; however, if nothing else in the community is changed, the disease can return eventually to its previous levels. Hence, health promotion and environmental improvements have pivotal role as a consolidating long-term interventions for marked reduction in active disease, which is thought to be an indicator for future blindness.

### Limitations of the study

In this study, the prevalence of active trachoma was determined by clinical finding not on microbiological identification of Chlamydia trachomatis. But some studies showed that clinical evaluation can be a useful tool to evaluate the response of azithromycin to active trachoma cases in a country with limited resources^9^. Though efforts were made to get accurate data, there could be a recall bias by parents as to how many times their children received azithromycin treatment in the past. Baseline data on the prevalence of active trachoma was not determined based on the WHO recommended trachoma survey methods, hence we used national survey result for comparison of our data.

## Conclusion

Though it was demonstrated that repeated mass oral azithromycin distributions has reduced active trachoma in the community, the prevalence of active trachoma in the district was not negligible; therefore, other trachoma specific control interventions such as fly control program, water supply changes, measures to improve face washing and construction of utilizable latrines that are being implemented by the health extension package has to be strengthened in the district. This essentially needs intersectoral collaboration between governmental organizations like the district health bureau and other bureau such as water development bureau, environmental protection/sanitation bureau and education bureau - strongly arguing for continued use of all the components of the SAFE strategy together in the community. Finally, as the current prevalence of TF was still more than 10%, we recommend azithromycin mass distribution in the community.

## Competing interests

The authors declare that they have no competing interests.

## Authors’ contributions

FA: conception and initiation of the study, design, implementation, analysis and drafting the manuscript. SB: design, implementation of the study and co-writing AB: design, implementation, analysis and co-writing. BA: analysis, interpret the data, co-writing and reviewed the manuscript. All authors have read and approved of the final version of the manuscript.

## Pre-publication history

The pre-publication history for this paper can be accessed here:

http://www.biomedcentral.com/1471-2431/13/199/prepub
